# Synergistic roles of CBX4 chromo and SIM domains in regulating senescence of primary human osteoarthritic chondrocytes

**DOI:** 10.1186/s13075-023-03183-8

**Published:** 2023-10-12

**Authors:** Yu-Hsiu Chen, Xin Zhang, David Attarian, Virginia Byers Kraus

**Affiliations:** 1 Duke Molecular Physiology Institute, Duke University, 300 N Duke St, Durham, NC 27701 USA; 2 Division of Rheumatology/Immunology/Allergy, Department of Internal Medicine, Tri-Service General Hospital, National Defense Medical Center, Taipei, Taiwan, ROC; 3 Department of Pathology, Duke University Medical Center, Durham, NC USA; 4 Department of Orthopaedic Surgery, Duke University, Durham, NC USA; 5 Department of Medicine, Duke University School of Medicine, Durham, NC USA

**Keywords:** CBX4, Cellular senescence, DPP4, Senomorphic, Osteoarthritis

## Abstract

**Background:**

Cellular senescence is a critical factor contributing to osteoarthritis (OA). Overexpression of chromobox homolog 4 (CBX4) in a mouse system was demonstrated to alleviate post-traumatic osteoarthritis (PTOA) by reducing cellular senescence. Additionally, replicative cellular senescence of WI-38 fibroblasts can be attenuated by CBX4. However, the mechanisms underlying this senomorphic function of CBX4 are not fully understood. In this study, we aimed to investigate the role of CBX4 in cellular senescence in human primary osteoarthritic chondrocytes and to identify the functional domains of CBX4 necessary for its function in modulating senescence.

**Methods:**

Chondrocytes, isolated from 6 individuals undergoing total knee replacement for OA, were transduced with wild-type CBX4, mutant CBX4, and control lentiviral constructs. Senescence-related phenotypic outcomes included the following: multiple flow cytometry-measured markers (p16^INK4A^, senescence-associated β-galactosidase [SA-β-gal] activity and dipeptidyl peptidase-4 [DPP4], and proliferation marker EdU), multiplex ELISA-measured markers in chondrocyte culture media (senescence-associated secretory phenotypes [SASPs], including IL-1β, IL-6, IL-8, TNF-α, MMP-1, MMP-3, and MMP-9), and PCR array-evaluated senescence-related genes.

**Results:**

Compared with control, CBX4 overexpression in OA chondrocytes decreased DPP4 expression and SASP secretion and increased chondrocyte proliferation confirming CBX4 senomorphic effects on primary human chondrocytes. Point mutations of the chromodomain domain (CDM, involved in chromatin modification) alone were sufficient to partially block the senomorphic activity of CBX4 (p16^INK4A^ and DPP4 increased, and EdU decreased) but had minimal effect on SASP secretion. Although having no effect on p16^INK4A^, DPP4, and EdU, deletion of two small-ubiquitin-like-modifier-interaction motifs (CBX4 ΔSIMs) led to increased SASP secretion (IL-1β, TNF-α, IL-8). The combination CBX4 CDMΔSIMs altered all these measures adversely and to a greater degree than the single domain mutants. Deletion of the C-terminal (CBX4 ΔC-box) involved with transcriptional silencing of polycomb group proteins increased IL-1β slightly but significantly but altered none of the other senescence outcome measures.

**Conclusions:**

CBX4 has a senomorphic effect on human osteoarthritic chondrocytes. CDM is critical for CBX4-mediated regulation of senescence. The SIMs are supportive but not indispensable for CBX4 senomorphic function while the C-box is dispensable.

**Supplementary Information:**

The online version contains supplementary material available at 10.1186/s13075-023-03183-8.

## Introduction

Osteoarthritis (OA) is the most common arthritis in adults; the worldwide prevalence of knee OA among individuals aged 15 or over approximates 16% [1]. The symptoms of OA include joint pain and stiffness, which can lead to decreased mobility and increased mortality [2, 3]. However, no disease-modifying anti-osteoarthritis drugs (DMOADs) are yet approved for the treatment of OA. Cellular senescence has been identified as a factor contributing to OA [4–6]. Cellular senescence is characterized by cell cycle arrest and the elicitation of senescence-associated secretory phenotypes (SASPs), which can impact surrounding cells and tissues [7]. Transplantation of senescent fibroblasts into knee joints of mice resulted in development of radiological and histological OA as well as pain and limited motion [8]. Furthermore, a previous study demonstrated that senescent cells accumulate in the cartilage and synovium in a post-traumatic OA (PTOA) mouse model; eliminating these senescent cells from the joint by a senolytic drug (UBX0101) prevented the development of PTOA in this mouse model [6]. These data support the supposition that suppression and regulation of senescence would have high therapeutic potential in OA.

Recently, CBX4 was shown to mitigate post-traumatic OA (PTOA) upon overexpression in a mouse system by reducing cellular senescence [9]. CBX4 is a polycomb repressive complex-1 (PRC1)-associated protein and a SUMO E3 ligase involved in cell cycle regulation and DNA damage repair [10, 11]. CBX4 contains three major functional domains: a chromodomain (CDM) at the N-terminus, small-ubiquitin-like-modifier (SUMO)-interaction motifs (SIMs), and a conserved chromobox (C-Box) at the C-terminus. The CDM is involved in chromatin modification and has been linked to the repression of *CDKN2A* (*p16*) expression [12, 13]. SIMs consisting of two motifs, SIM1 and SIM2, are responsible for the SUMO E3 ligase function. They facilitate DNA damage repair through BMI1 recruitment regulation [11, 14]. The C-box contributes to the transcriptional silencing function of polycomb group (PcG) proteins by recruiting other PcG proteins to target genes [10]. CBX4 is a repressor of C-MYC, while deletion of the C-box results in enhanced expression of this proto-oncogene [15]. CBX4 upregulation has previously been shown to correlate with tumor growth and metastasis in breast cancer and hepatocellular cancer [16, 17]. In our previous study, we demonstrated that CBX4 protein expression of WI-38 primary human fibroblasts decreased with serial cell replication in culture [18]. Moreover, in association with reduced CBX4 levels, senescence markers increased, whereas activating CBX4 was associated with a decrease of these markers [18]. These findings suggest that CBX4 regulates replicative senescence in WI-38 cells and functions as a senomorphic and potential anti-senescence target; however, direct mechanisms of its senomorphic effect are lacking.

We hypothesized that CBX4 is a senomorphic with direct effects on chondrocyte senescence. To test our hypothesis, we evaluated the change in senescence phenotype and cell proliferation of primary human OA chondrocytes using several widely used markers including p16^INK4A^, senescence-associated β-galactosidase (SA-β-gal) activity, and EdU (5-ethynyl-2′-deoxyuridine) [19]. Additionally, we included dipeptidyl peptidase-4 (DPP4) that we recently identified as a cell surface marker of chondrocyte senescence in OA [20]. We confirmed the senomorphic effect of CBX4 in OA joint tissue by overexpressing CBX4 in chondrocytes isolated from knee joints of OA patients. In addition, through analyses of full-length wild type (WT) and mutant CBX4 constructs carrying loss of function mutations in one of the three known primary functional domains of CBX4, we identified functional domains contributing to the senomorphic effects of CBX4 in primary chondrocytes.

## Materials and methods

### Primary chondrocyte isolation and culture

Human knee joint tissues were obtained from OA patients during total knee arthroplasty as surgical waste under IRB approval of Duke Hospital (*n* = 6). Human articular cartilage from the tibial plateau and femoral condyle (both lesioned and non-lesioned) was finely diced and digested in pronase 0.1% (weight and volume, w/v, Roche, 10165921001) for 1 h, followed by 0.17% (w/v) type II collagenase (Sigma, C6885) in chondrocyte culture media for 16–18 h yielding a mean (± standard error of mean, SEM) 5.48 ± 1.01 × 10^6^ chondrocytes per gram of human articular cartilage [20]. Chondrocyte media contained DMEM/F-12, GlutaMAX™ (Thermo Fisher, 10565018) with 10% heat-inactivated fetal bovine serum (HI FBS, Thermo, 10082147), Penicillin–Streptomycin 1x (Thermo, 15140122), and 50 μg/ml l-ascorbic acid (Sigma-Aldrich, A8960). Isolated chondrocytes were seeded at a density of 200,000 cells/well in 24 well plates. The primary chondrocytes were cultured in a monolayer for 5 days in chondrocyte culture media and transduced with empty vector control, WT CBX4, or the mutant CBX4 in lentiviral constructs.

### Lentiviral particle preparation

Lentiviral particles used for transduction were prepared by VectorBuilder (Chicago, Illinois) (Table 1). mCherry expressing lentiviral particles were used as a control to monitor and optimize transduction efficiency. Wildtype CBX4 cDNA (NM_003655.3) and mutants were cloned into pLV-Puro-CMV with lentivirus packaging; pLV-Puro-CMV with lentivirus packaging was used as a control. CDM-mutated CBX4 (CBX4 CDM) was created with F11A and W35L mutants [12]. SIM-deleted CBX4 (CBX4 ΔSIMs) was created with both SIM1 (amino acids 262–265) and SIM2 (amino acids 462–465) deletion [10]. C-box-deleted CBX4 (CBX4 ΔC-box) was created with C-box (amino acids 531–556) deletion [21]. A combination CDM and SIM mutant was also created (CBX4 CDMΔSIM).

**Table 1:** CBX4-related lentiviral constructs used for viral transduction of primary human chondrocytes

Created vector	Vector ID	Vector name	Size	Function of domain
Wild-type CBX4 (CBX4 WT)	VB-210801-1043rpz	pLV[Exp]-Puro-CMV > hCBX4[NM_003655.3]	9588 bp	
CDM-mutated CBX4 (CBX4 CDM)	VB-210709-1084wpn	pLV[Exp]-Puro-CMV > hCBX4*(F11A, W35L)	9588 bp	PRC1-related chromatin modification
SIM-deleted CBX4 (CBX4 ΔSIMs)	VB-210709-1077tkm	pLV[Exp]-Puro-CMV > hCBX4*(delete 262–265, 462-465aa)	9564 bp	SUMO E3 ligase function
C-box-deleted CBX4 (CBX4 ΔC-box)	VB-210709-1085knf	pLV[Exp]-Puro-CMV > hCBX4*(delete 531-556aa)	9510 bp	Transcriptional silencing function of pcG proteins
CDM-mutated and SIM-deleted CBX4 (CBX4 CDMΔSIM)	VB-210709-1088mgj	pLV[Exp]-Puro-CMV > hCBX4* (F11A, W35L; delete 252–265,462-465aa)	9564 bp	
Control (Ctrl)	VB-210808-1067mmj	pLV[Exp]-Puro-CMV	7899 bp	
mCherry control	VB010000-9298rtf	pLV[Exp]-EGFP:T2A:Puro-EF1A > mCherry	10085 bp	

### Transduction of lentiviral particles

The addition of mCherry control lentiviral articles at a multiplicity of infection (MOI) of 20, along with spinfection [22] at 900 × g for 1 h, resulted in approximately 60% mCherry expression in primary chondrocytes by 3 days after transduction (data not shown). Based on the mCherry control result, we estimated a 60–70% transduction efficiency by CBX4 WT, CBX4 mutant, and control lentiviral constructs in chondrocytes. To mitigate the risk of viral toxicity linked to higher MOI, and avoid inducing senescence through polybrene-related cellular stress [18, 23], we chose not to increase the MOI beyond the selected level and to avoid employing polybrene. CBX4 WT, CBX4-mutated, and control lentiviral constructs were added to primary chondrocytes using spinfection 900 × g for 1 h with MOI 20. After transduction, the chondrocytes were cultured for 5 days. The culture media were replaced 24 h after transduction, and then again 4 days later. After the second media replacement, the culture media were collected for a period of 24 h. Subsequently, EdU (Thermo Fisher, C10424) was added to the chondrocyte culture media at a concentration of 10 μM. After 24 h of incubation with EdU, the cells were collected for further analysis.

### Flow-cytometric analysis of senescence markers

Chondrocytes were assessed for cellular senescence status by quantification of DPP4 and p16^INK4A^ protein expression, and SA-β-gal activity using flow cytometry as we previously described [18, 20]. For quantification of cell surface DPP4, 1–2 × 10^4^ cells were stained with anti-DPP4 monoclonal antibody (2A6), PE (Thermo Fisher, 12–0269-42; 2 µl/100 µl) in phosphate-buffered saline (PBS, Thermo, 10010023) with 1% bovine serum albumin (BSA, Sigma A3294) for 30 min at room temperature, and then washed twice with 600 µl PBS with 1% BSA. Co-staining of SA-β-gal activity with the cell proliferation marker EdU), and senescence marker p16^INK4A^, was performed as we previously described [18]. Briefly, after cell fixation with 4% paraformaldehyde (PFA, Thermo Fisher 50980487), SA-β-gal activity was quantified using the CellEvent Senescence Green flow cytometry assay (Thermo Fisher, C10840) carried out at 37 °C for 2 h per the manufacturer’s instructions. The cells were then permeabilized with permeabilization buffer (Thermo Fisher, 00–8333-56) and stained for p16^INK4A^ or EdU. Anti-p16 mAb (20:100, Roche CINtec kit 9517) was used for p16^INK4A^ staining for 30 min followed by washing and 30 min incubation with AF647-conjugated anti-mouse IgG2a secondary antibody (1:1000, Jackson, 115607186). The Click-iT™ EdU Alexa Fluor™ 647 flow cytometry assay (Thermo Fisher, C10424) was used to identify EdU positive cells following the manufacturer’s instructions. The unstained and stained cells were analyzed using an Attune NxT flow cytometer (Thermo Fisher). Unstained cells were used to determine the fluorescence background. Data were analyzed using the FlowJo V10.8 software (BD Life Sciences).

### Western blot

Western blot was performed as previously described [18]. Primary chondrocyte cell lysis and protein extraction were achieved with the PARIS™ kit (Thermo Fisher AM1921) with proteinase inhibitors (Sigma-Aldrich, P8340). A total 5 μg of protein lysates was mixed with 4 × Laemmli sample buffer (Bio-Rad 1610747) and heated to 95 °C for 5 min. Cell lysates (5 μg/well, Bio-Rad#4568033) were separated on a 10% Mini-PROTEAN® TGX Stain-Free™ protein gels (Bio-Rad, 4568033). Proteins were transferred to polyvinylidene fluoride (PVDF) membranes using the Trans-blot turbo system (Bio-Rad,#1704274). The membrane was blocked with 5% w/v fat-free milk (CST 9999 s) for 1 h at room temperature. Membranes were washed three times with Tris-buffered saline Tween 20 buffer (TBST, Thermo Fisher, 28360) then incubated with the primary antibody, anti-CBX4 mAb (Cell Signaling Technology #30559, 1:1000), or internal control, anti-β-actin mAb-HRP (Santa Cruz Bio, sc-47778 HRP, 1:2000), in TBST containing 5% BSA at 4° C overnight. According to the manufacturer, the anti-CBX4 mAb (Cell Signaling Technology #30559) is produced by immunizing animals with a synthetic peptide corresponding to residues surrounding Pro166 of human CBX4 protein, which should be able to detect all the CBX4 mutants generated. The following day, membranes were washed three times with TBST and incubated with anti-rabbit IgG-HRP (1:500, Thermo Fisher 32460) at room temperature for 1 h. β-actin protein bands were visualized using Clarity™ Western ECL Substrate (Bio-Rad, 1705060); CBX4 protein bands were visualized using SuperSignal™ West Pico PLUS Chemiluminescent Substrate (Thermo Fisher 34579). Membrane Images were acquired with the ChemiDoc XRS system (Bio-Rad, USA). Grey band density values were quantified using Image lab (version 6.0, Bio-Rad).

### Senescence-associated secretory phenotypes

IL-1β, IL-6, IL-8, and TNF-α were quantified by a V-PLEX Human Proinflammatory Panel II 4-Plex immunoassay (MSD, K15053D) with intra- and inter-assay coefficients of variation (CVs) 3.8%, 4.0%, 3.1%, 2.8% and 6.4%, 6.4%, 6.4%, and 8.0%, respectively. Matrix metalloproteinase (MMP)-1, MMP-3, and MMP-9 were quantified by a Human MMP 3-Plex Ultra-Sensitive Kit (MSD, K15034C) with intra-assay CVs 4.0%, 3.4% and 2.3%, respectively, and inter-assay CVs ≤ 18%.

### Quantitative real-time polymerase chain reaction (qRT-PCR) and qRT-PCR array

RNA was isolated from transduced primary chondrocytes using a PARIS™ Kit (Thermo Fisher AM1921). cDNA was synthesized using the iScript™ cDNA Synthesis Kit (Bio-Rad, 1708891). Subsequently, qRT-PCR was performed using an SYBR green master mix (Applied Biosystems, 4309155) with a QuantStudio 6 Flex Real-Time PCR System (Applied Biosystems). Gene expression of CBX4 was measured using YWHAZ as an internal reference control gene. Primer sequences were as follows: CBX4 Forward ACCGTGCCAAGCTGGATTT; CBX4 Reverse AGGTCGTACATTTTGGGGTCG; YWHAZ Forward CTGAGGTTGCAGCTGGTGATGACA; YWHAZ Reverse AGCAGGCTTTCTCAGGGGAGTTCA.

A custom RT2 Profiler PCR Array (Qiagen, 330171) was employed to analyze the expression levels of 42 specific genes associated with cellular senescence and/or CBX4 as previously described (Table S1) [18]. The RT^2^ First Strand Kit (Qiagen, 330404) was utilized to synthesize cDNA. Subsequently, qRT-PCR was performed using a RT^2^ SYBR Green ROX qPCR Mastermix (Qiagen, 330522) with the QuantStudio 6 Flex Real-Time PCR System (Applied Biosystems) according to the manufacturer’s instructions. The CT value of each gene was normalized with reference gene *YWHAZ*, ΔCT = CT_target gene_ − CT_*YWHAZ*_. The relative gene expression was calculated by ΔΔCT (ΔCT _CBX4 mutant_ − ΔCT _CBX4 WT_), which was used to calculate the relative expression ratio (2^−ΔΔCT^). Genes with over 40% of values undetected (CT > 40) were excluded; for any sample with a gene CT value undetected, CT 40 was imputed. *DAO*, *TP63*, *LY6D*, and *SOX2* were not detected; *CBX4* was included in the PCR array, but the primer used was not able to detect all the mutants. For these reasons, we excluded these five genes from analysis resulting in a total 37 genes analyzed.

### Statistical analysis

Data are presented as mean ± SEM. Analyses were performed using Prism 9 (GraphPad software). Flow cytometry measurements and SASP secretion by primary chondrocytes transduced by either CBX4 WT or control passed the lognormal Shapiro–Wilk and Kolmogorov–Smirnov distribution tests. Following this assessment, ratio-paired *t*-tests were conducted to further analyze the data. Repeated measures with Dunnett’s post hoc test were performed for comparison of WT CBX4 with each of the mutants transduced into chondrocytes; the data for p16^INK4A^, EdU, IL-1β, and TNF-α exhibited violations of sphericity, necessitating use of a Geisser-Greenhouse correction when calculating the *P*-values for repeated measures ANOVA. *P* < 0.05 was considered statistically significant. Benjamini and Hochberg false discovery rate (FDR) < 0.1 and a log2 fold change (FC) > 0.58 thresholds were applied to the qRT-PCR array data analysis.

## Results

### CBX4 overexpression in primary OA chondrocytes decreased senescence and inflammatory response and increased cell proliferation in primary OA chondrocytes

**Fig. 1:**
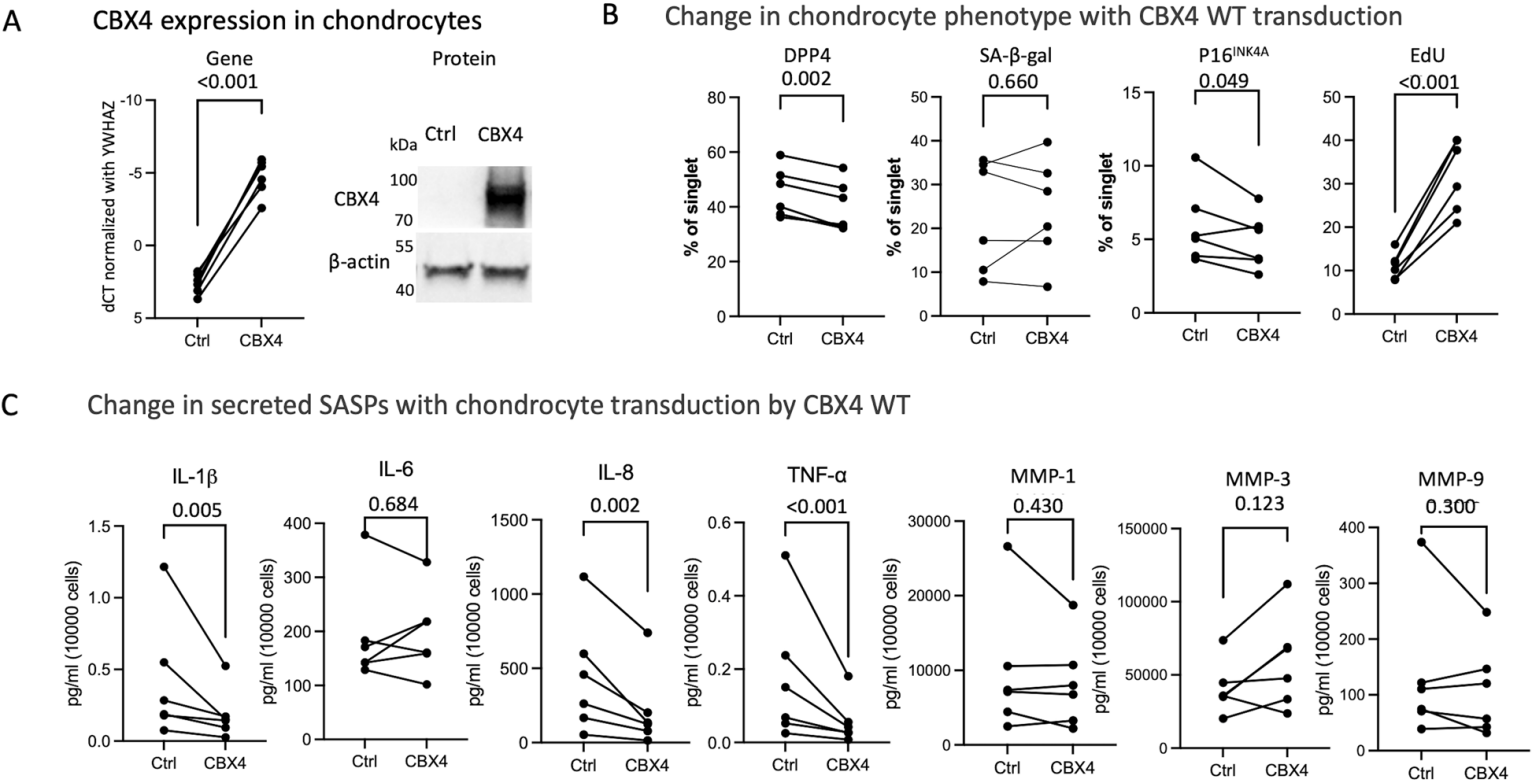
Effects of CBX4 on primary human chondrocytes. CBX4 was overexpressed in human OA chondrocytes using CBX4 WT lentiviral particles. **A** CBX4 gene expression (*n* = 6) and protein expression (representative Western blot) in the CBX4 WT and control lentiviral construct (Ctrl) transduced OA chondrocytes. Upon longer exposure of the Western blot, CBX4 protein expression in control transduced primary chondrocytes was comparable to levels of expression we observe in senescent WI-38 (passage 55) cells (data not shown). CBX4: 75 kDa, β-actin: 42 kDa. **B** SA-β-gal activity, protein expression of p16^INK4A^ and DPP4, and EdU proliferation were detected using flow cytometry. **C** Senescence-associated secretory phenotype (SASP) secretion was measured in the culture supernatants of CBX4 WT and vector only control (Ctrl)-transduced OA chondrocytes. Ratio-paired *t*-tests were performed to compare control and CBX4 WT presented as a dot line graph; *P* values are listed in the figure

To investigate the impact of CBX4 on primary chondrocyte senescence status, we successfully and dramatically overexpressed CBX4 gene and protein expression with CBX4 WT transduction. Compared with control lentiviral construct transduced chondrocytes, the mean gene expression (ΔCT) of CBX4 in primary transduced chondrocytes from CBX4 WT was 159-fold higher (*P* < 0.0001). Similarly, an intense band corresponding to CBX4 protein was identified from the lysate of CBX4 WT-transduced chondrocytes, while no band was detected from the control (vector transduction) cell lysate using the same blot exposure time (3 s). With longer exposure time (40 s), native CBX4 protein expression was detected in chondrocytes transduced with the control lentiviral construct; native CBX4 amount corresponded to levels we observe in comparable amounts of a protein lysate derived from WI-38 senescent cells (data not shown) (Fig. 1A).

Compared with control cells, CBX4 WT-transduced chondrocytes had a significantly lower mean frequency of DPP4^+^ and p16^INK4A+^ cells and a significant (> threefold) increase in mean cell proliferation based on the EdU^+^ signal (Fig. 1B). CBX4 WT-transduced chondrocytes secreted significantly less mean IL-8, IL-1β, and TNF-α (Fig. 1C). These findings suggest that CBX4 WT transduction resulted in a senomorphic effect characterized by lower expression of senescence markers, including SASP secretion.

### Changes in senescence chondrocyte phenotype in response to CBX4 wild-type and mutants

**Fig. 2:**
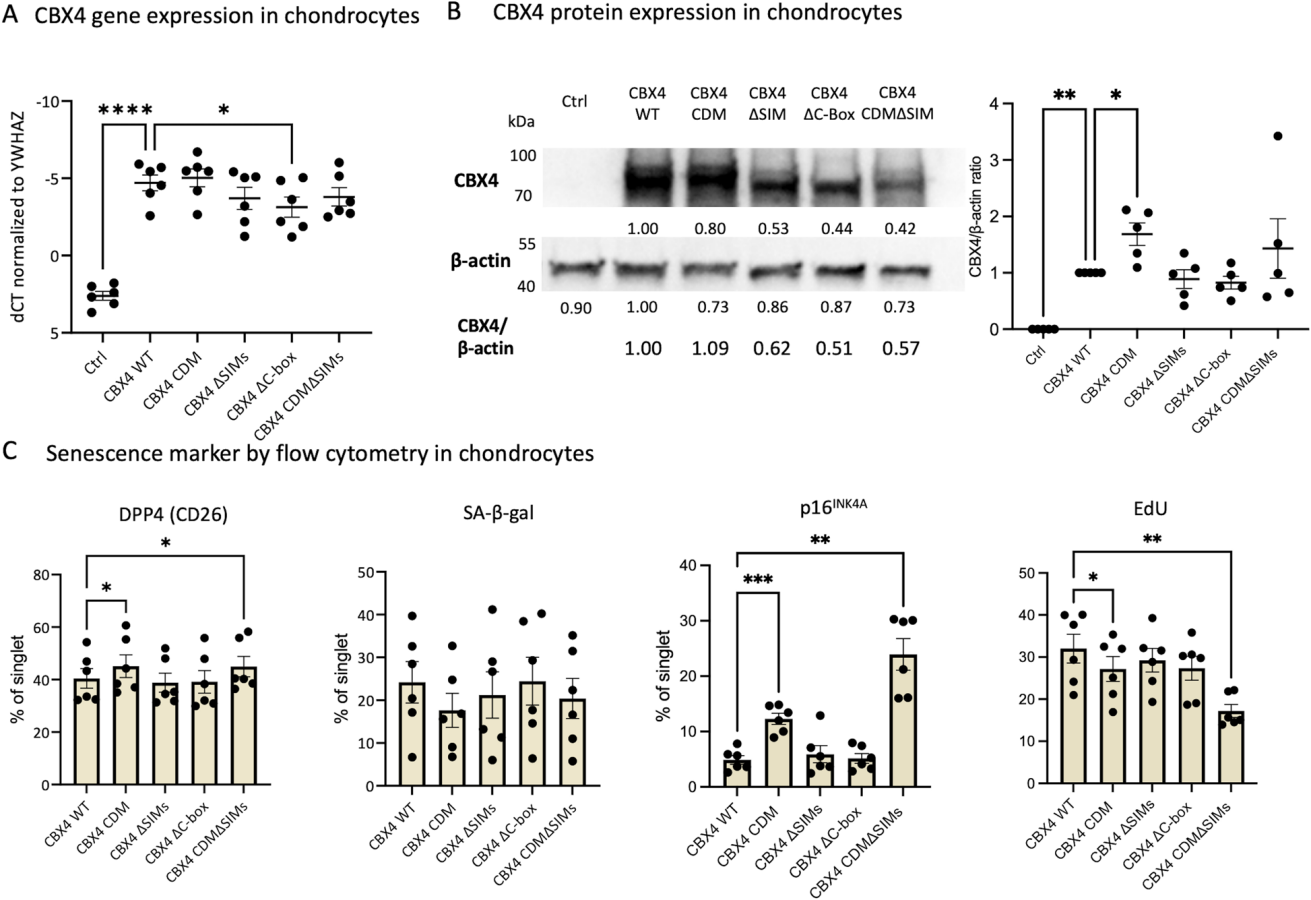
Mapping effects of CBX4 (wild-type and mutants) on primary human chondrocytes. Expression of senescence markers and cell proliferation of primary human chondrocytes transduced by CBX4 wild-type (WT) or mutants. **A** CBX4 gene expression (*n* = 6) and **B** protein expression by Western blot (representative blot with quantification of *n* = 5 biological replicates in dot plot) in cells transduced by the empty vector Control (Ctrl), CBX4 WT, or its mutants. The expected molecular weights of CBX4 WT, CBX4 CDM, CBX4 ΔSIMs, CBX4 ΔC-box, and CBX4 CDMΔSIM were 75 kDa, 75 kDa, 74 kDa, 72 kDa, and 74 kDa, respectively. **C** Protein-level expression of senescence markers DPP4, SA-β-gal activity, and p16.^INK4A^ and cell proliferation marker EdU quantified by flow cytometry and plotted as a dot bar graph. Repeated measures with Dunnett’s post hoc test were performed to compare the effects of CBX4 WT and mutants. *P ≤ 0.05, **P ≤ 0.01, ***P ≤ 0.001, ****P < 0.0001

In human primary OA chondrocytes, we successfully overexpressed CBX4 WT and CBX4 mutants with mutations of the individual major functional domains of CBX4. The mean gene expression (ΔCT) and protein expression of CBX4 from CBX4 WT and all tested CBX4 mutants (CBX4 CDM, CBX4 ΔSIMs, CBX4 ΔC-box, and CBX4 CDMΔSIMs) were dramatically higher than control (Fig. 2A, B). Additionally, compared with CBX4 WT transduction, CBX4 ΔC-box had lower CBX4 gene expression (Fig. 2A), while CBX4 CDM had higher CBX4 protein expression (Fig. 2B).

Compared with CBX4 WT, despite the highest-level expression of all the constructs, the CBX4 CDM mutant increased senescence as evidenced by a significantly higher mean percentage of chondrocytes expressing senescence-associated biomarkers, DPP4 and p16^INK4A^, but a significantly lower mean percentage of EdU^+^ proliferating cells (Fig. 2C). The individual CBX4 ΔSIMs and CBX4 ΔC-box mutants did not affect these senescence outcomes. Compared with CBX4 CDM, the CBX4 CDMΔSIM combination mutant further increased mean p16^INK4A^ and further reduced cell proliferation. These data show that CDM is critical for CBX4-mediated regulation of senescence, while the SIMs are supportive but not indispensable for CBX4 senomorphic function (Fig. 2C).

**Fig. 3:**
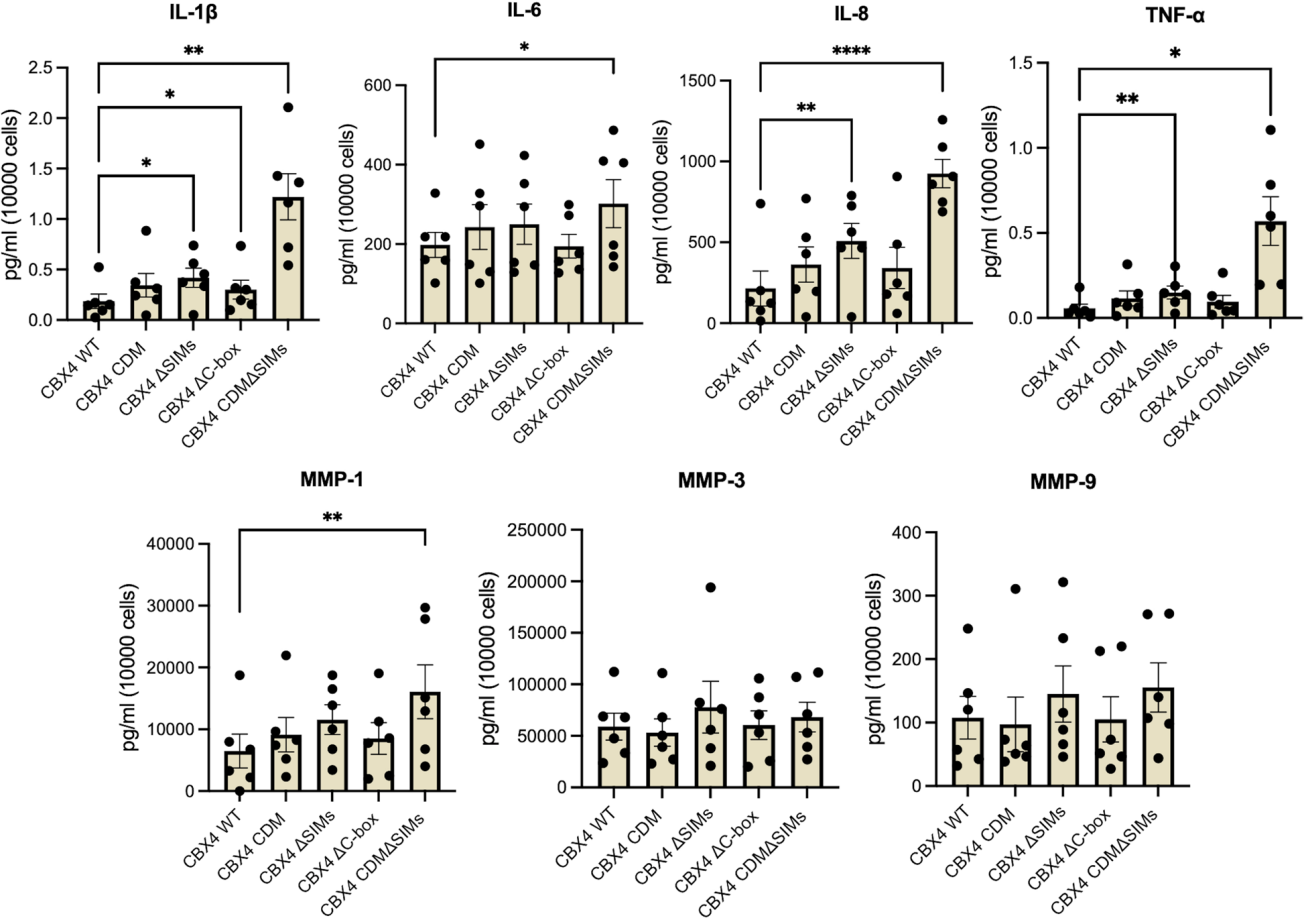
Effects of CBX4 wild-type and mutants on SASP secretion by primary human OA chondrocytes. Comparison of SASP secretion, including IL-1β, IL-6, IL-8, TNF-α, MMP-1, MMP-3, and MMP-9, by primary human OA chondrocytes transduced with CBX4 wild-type (WT) or mutants. Repeated measures with Dunnett’s post hoc test were performed to compare WT and mutant CBX4. *P ≤ 0.05, **P ≤ 0.01, ***P ≤ 0.001, ****P ≤ 0.0001

Next, we investigated SASP secretion by OA chondrocytes overexpressing CBX4 WT and CBX4 mutants. Compared with CBX4 WT, chondrocytes transduced with the combination CBX4 CDMΔSIM mutant secreted significantly more IL-1β, IL-6, IL-8, TNF-α, and MMP-1 (Fig. 3, *n* = 6). Compared to CBX4 WT, the individual CBX4 ΔSIM mutant also secreted significantly more IL-1β, IL-8, and TNF-α. In addition, compared with wild type control, we observed a significant increased IL-1β secretion by the ΔC-box and marginally increased secretion by the CDM mutation. These SASP findings demonstrate that the CBX4 SIM domains in large part regulate SASP secretion in primary chondrocytes. Thus, the combination CBX4 CDMΔSIMs altered all the measures (primary senescence markers and SASPs) adversely and to a greater degree than the single domain mutants demonstrating the synergy of these domains for CBX4 senomorphic functions.

### Senescence-related PCR-array analysis in response to CBX4 overexpression

**Fig. 4:**
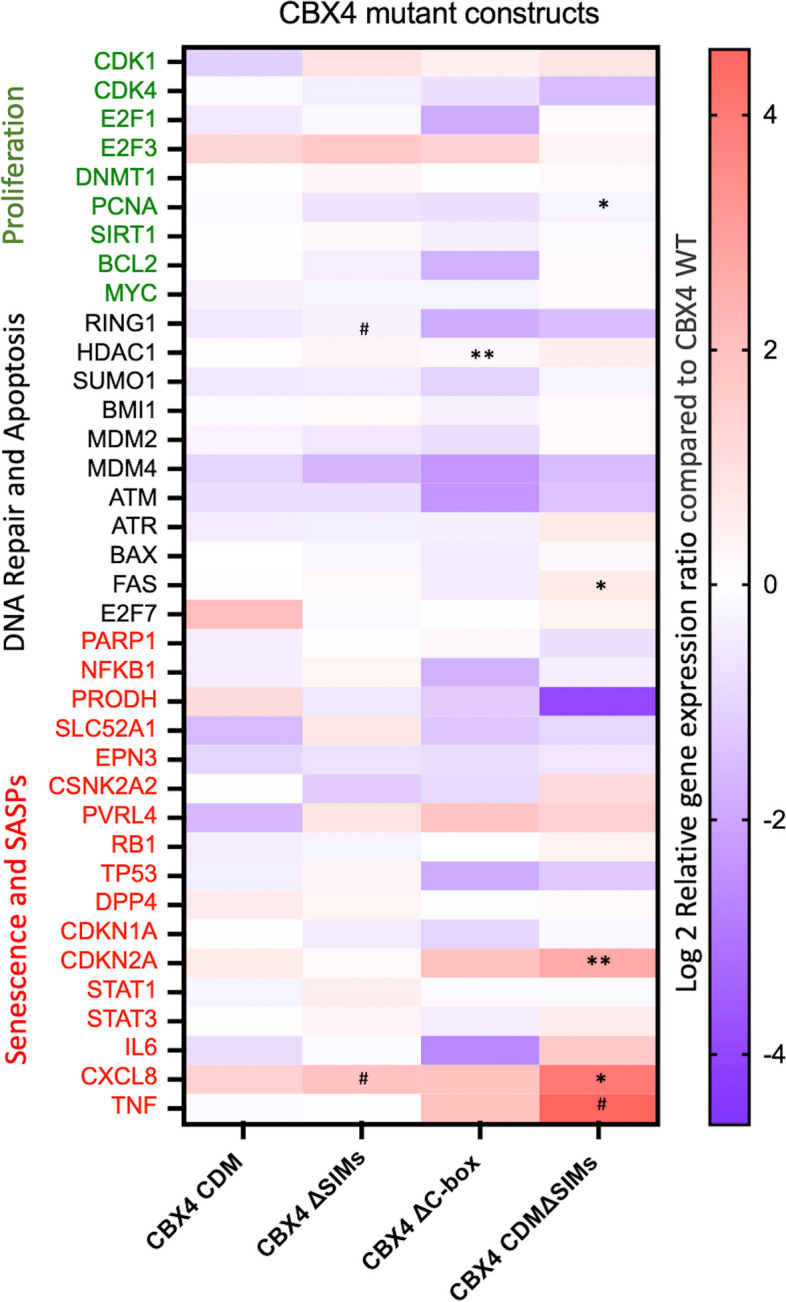
Senescence-related gene expression profiling of CBX4 wild-type and mutants in primary human OA chondrocytes. The heatmap depicts the relative gene expression by primary human OA chondrocytes transduced with CBX4 wild-type (WT) or four CBX4 mutants of a select group of genes quantified by qPCR array. Gene expression is shown as a log2 ratio of expression level by CBX4 mutant-transduced cells relative to the CBX4 WT-transduced condition. The genes in the heatmap are organized vertically based on their functional properties: green text labels (top *y*-axis) are genes associated with cell proliferation; black text labels (middle *y*-axis) are genes related to DNA damage repair and apoptosis; red text labels (bottom *y*-axis) are genes related to SASPs (senescence-associated secretory phenotypes) and cellular senescence. The most deleterious impact on CBX4 senomorphic capability was caused by the combination of CDM and SIM mutations (far right column). ^#^*P* ≤ 0.1 *P ≤ 0.05, **P ≤ 0.01

Analysis of expression of a total of 37 selected genes revealed that the most deleterious impact on CBX4 senomorphic capability in primary human OA chondrocytes was observed with the CBX4 CDMΔSIM mutant, i.e., the combination of CDM and SIM mutations: increased apoptosis inducer *FAS* (*P* = 0.03), increased senescence gene *CDKN2A* (*P* = 0.003), increased inflammatory chemokine *CXCL8* (*P* = 0.02), and marginally increased SASP, *TNF* (*P* = 0.09), and decreased cell proliferation-related gene *PCNA* (*P* = 0.02) (Fig. 4). In addition, compared with CBX4WT, CBX4 ΔC-box significantly increased *HDAC1* (*P* = 0.006) known to restrict transcription initiation frequency [24] and transcriptional elongation [25]. CBX4 ΔSIMs marginally increased *CXCL8* (*P* = 0.07) and decreased expression of the transcriptional repressor, *RING1* (*P* = 0.08) (Fig. 4). Based on FDR < 0.1 and log2 FC > 0.58, the combination of CDM and SIM mutations of CBX4 significantly increased *CDKN2A* and* CXCL8.*

## Discussion

In this study, we demonstrated the senomorphic effect of CBX4 overexpression in human OA chondrocytes, based on decreased DPP4 expression, p16^INK4A^ and SASP secretion, and increased proliferation indicated by the EdU assay. Our findings strongly support a senomorphic role of CBX4 in OA chondrocytes and the potential for exogenous CBX4 as a senomorphic treatment in OA. In addition, we investigated the role of CDM, SIM motifs, and C-box domains of CBX4 in regulating senescence of primary OA chondrocytes. Double point mutations of the CDM domain alone were enough to partially block the senomorphic activity of CBX4. The deletion of SIMs alone enhanced secretion of multiple SASPs, without significantly affecting any of the other assessed parameters indicative of senescence in OA chondrocytes. However, CDM mutations together with deletion of the two SIM domains, the CDMΔSIM mutant, synergistically blocked the senomorphic effects of CBX4, as demonstrated by increased senescence-related protein and/or gene expression (DPP4, p16^INK4A^/*CDKN2A*, *CXCL8*), reduced cell proliferation (EdU), and increased SASP secretion (IL-1β, IL-6, IL-8, TNF-α, and MMP-1). Although the CBX4 ΔC-box significantly decreased IL-1β secretion, deletion of this domain did not significantly impact any of the other indicators of OA chondrocyte senescence. Our findings suggest that the CDM domain is essential for CBX4 senomorphic function, while the SIM domains play a secondary or modulatory role with a primary impact on SASP secretion.

We investigated the effect of CBX4 on chondrocyte senescence using several markers, including a newly identified marker, DPP4, whose expression on the surface of OA chondrocytes we showed is associated with higher cellular secretion of SASP factors [20]. Compared with control, CBX4 overexpression in human OA chondrocytes significantly decreased DPP4 expression, p16^INK4A^ expression, and SASP secretion. These observations are consistent with our prior observations in WI-38 cells in which DPP4 expression and p16^INK4A^ expression were upregulated and downregulated by CBX4 decrease and increase, respectively [18], suggesting that CBX4 may modulate senescence in chondrocytes, in part, through regulation of DPP4 and p16^INK4A^. The CDM (chromodomain) of CBX4 is known to repress the expression of cyclin-dependent kinase inhibitors, such as p16^INK4a^ and p14/p19^ARF^, through the action of PRC1 [26, 27]. Previous research has shown that PRC1 binds to the *p16* promoter and represses its expression in young cells [28]. It is yet to be determined whether DPP4 is a direct target and/or mediator of CBX4 regulation of senescence.

Although CBX4 overexpression led to significant decreases in p16^INK4A^ and DPP4, it did not result in a notable reduction of SA-β-gal levels. This observation could be a result of the intricate regulation of senescence pathways, with CBX4 potentially influencing some pathways more strongly than others. Other factors, including distinct threshold requirements for detection and heterogeneous cellular responses, could also contribute to this phenomenon. In addition, senescence is a dynamic process that can involve both early and late events. The observed changes might be time-dependent, and the assessment might not have captured the optimal time point for observing changes in SA-β-gal levels. Future investigations, such as employing a stressor-induced model to potentially elevate SA-β-gal activity expression beyond the threshold, or considering different time points and alternative assays, might provide insights into the specific effects of CBX4 on senescence.

The SIM motif is related to the function of SUMO E3 ligase of CBX4. Sumoylation is a post-translational modification process where a member of the SUMO family of proteins is covalently attached to other proteins, thereby modifying their function [29]. A prior study showed that CBX4-mediated SUMO-modification was crucial for DNA damage repair by BMI1 [11]. Moreover, CBX4 can auto-sumoylate C-terminal binding protein, which is responsible for gene repression [30], and facilitate its own function [10]. Sumoylation has recently been found to be involved in senescence regulation, and the sumoylation of different proteins can have varied implications in senescence development [29]. For instance, sumoylation of SIRT1 promotes the survival of normal and cancer cells [31, 32], while sumoylation of protein peroxiredoxin 6 impairs its cell protective function [33]. We speculate that deletion of SIMs of CBX4 may impair DNA damage repair and increase expression of genes involved in senescence regulation. The CBX4 CDMΔSIM showed the greatest deleterious impact on senomorphic outcomes. The SIM deletions were mostly harmful in the context of the CDM mutations. The observed phenomenon can be attributed to the fact that CDM primarily regulates cell cycle checkpoint control. Thus, the mutation of CDM directly leads to cellular senescence. Following cellular senescence, the absence of SIMs further exacerbates the senescence phenotypes. However, SIM deletion is mainly associated with DNA damage repair. In this non-stress model system, the impact of SIM deletion was observed to relate primarily to SASP secretion.

We observed decreased transcript expression in CBX4 ΔC-box compared to CBX4 WT-transduced chondrocytes. The C-box is crucial to the transcriptional silencing role of polycomb group (PcG) proteins, attracting other PcG proteins to target genes. Deletion of the CBX4 C-box can diminish its transcriptional activity, reflecting the intricate functions of PcG proteins; they repress genes through polycomb repression complexes (PRCs) and histone modifications, while also activating transcription via interactions with elements such as transcription factors, non-coding RNAs, and post-translational modifications [34].

There were several limitations of this study. While CBX4 WT transduction exhibited a senomorphic effect in OA chondrocytes, the intrinsic expression levels of endogenous CBX4 in normal tissue relative to OA tissue remain uncertain. Our prior work suggests a modest upregulation of CBX4 in senescent (DPP4^+^) primary human chondrocytes that are enriched in OA-lesioned cartilage that is insufficient to fully counteract their senescence phenotype [20]. A comparative analysis between OA and normal cartilage or distinct regions within OA tissue could reveal valuable insights into the role of CBX4 in maintaining cartilage homeostasis and processes related to OA. To identify the effect of CDM, SIM, and C-box in senescence regulation, we tested each domain’s function separately. We did not test all combinations of mutants, only CDM mutations in combination with the SIM deletions; a broader combination could further the understanding of the interaction of the functional domains of CBX4. Moreover, the observed impact of various CBX4 mutations may be different under cell stress conditions, particularly for SIMs. We transduced human OA chondrocytes in bulk with CBX4 without antibiotic selection; thus, we evaluated the mean senomorphic outcomes of the total cell population; therefore, these results may underestimate the overall senomorphic effects of CBX4 and the impact of mutating its functional domains that might have been discerned with pre-selection of an at-risk chondrocyte population (e.g., senescent) or specific analysis of the cells that were effectively transduced. We nevertheless achieved a relatively high transduction efficiency; therefore, we believe these results to be generalizable to the biological effects that CBX4 is likely to have in vivo.

## Conclusions

In summary, we showed that CBX4 has a senomorphic effect on human osteoarthritic chondrocytes as evidenced by decreased DPP4, p16^INK4A^, and SASP secretion and increased cell proliferation. Additionally, we found a predominant effect of the CDM (chromodomain) and synergy with the SIM domains with respect to CBX4 regulation of chondrocyte senescence. CDM is mostly involved in maintaining cell cycle proliferation by decreasing p16^INK4A^ expression. The SIMs are involved in blocking SASP secretion and enhancing DNA damage repair. Taken together, we conclude that CBX4 is a multi-functional protein whose domains play synergistic roles in senescence regulation in human chondrocytes.

### Supplementary Information

**Additional file 1:** **Table S1.** qPCR custom microarray panel (Qiagen, 330171). **Supplementary Figure 1.** The Western blot underwent a selective exposure with a cutoff at 70 kDa, reflecting substantial variations in protein expression levels between CBX4 and β-actin. In Fig. 1A, the blot was further tailored to highlight regions specific to the Control (Ctrl) and CBX4 Wild Type (WT) samples; Fig. 2B, focused on CBX4 WT and its mutants within the molecular weight range of 70-100 kDa for CBX4 and 40-55 kDa for β-actin.

## Data Availability

All data generated or analyzed during this study are included in the published article.

## Abbreviations

OA: Osteoarthritis
CBX4: Chromobox homolog 4
SA-β-gal: Senescence-associated β-galactosidase
DPP4: Dipeptidyl peptidase-4
SASPs: Senescence-associated secretory phenotypes
CDM: Chromodomain
SUMO: Small ubiquitin-like modifier
SIMs: SUMO interaction motifs
C-box: Chromobox
DMOADs: Disease-modifying anti-osteoarthritis drugs
PTOA: Post-traumatic osteoarthritis
PRC1: Polycomb repressive complex -1
PcG: Polycomb group
EdU: 5-Ethynyl-2′-deoxyuridine
WT: Wild type
w/v: Weight/volume
HI FBS: Heat-inactivated fetal bovine serum
SEM: Standard error of mean
CBX4 CDM: CDM-mutated CBX4
CBX4 ΔSIMs: SIM-deleted CBX4
CBX4 ΔC-box: C-box-deleted CBX4
CBX4 CDMΔSIM: A combination CDM and SIM mutant
Ctrl: Control
MOI: Multiplicity of infection
PFA: Paraformaldehyde
CV: Coefficients of variation
FDR: False discovery rate
