# Supplementary material for: Synergistic roles of CBX4 chromo and SIM domains in regulating senescence of primary human osteoarthritic chondrocytes
Source: Arthritis Res Ther. 2023 Oct 12;25:197. doi: 10.1186/s13075-023-03183-8 (PMC10568837; doi:10.1186/s13075-023-03183-8)
Supplement: Supplementary file 1 — Additional file 1: Table S1. qPCR custom microarray panel (Qiagen, 330171). Supplementary Figure 1. The Western blot underwent a selective exposure with a cutoff at 70 kDa, reflecting substantial variations in protein expression levels between CBX4 and β-actin. In Fig. 1A, the blot was further tailored to highlight regions specific to the Control (Ctrl) and CBX4 Wild Type (WT) samples; Fig. 2B, focused on CBX4 WT and its mutants within the molecular weight range of 70-100 kDa for CBX4 and 40-55 kDa for β-actin. [file 13075_2023_3183_MOESM1_ESM.docx]

**SUPPLEMENTARY MATERIALS**

**Synergistic roles of CBX4 chromo and SIMs domains in regulating senescence of primary human osteoarthritic chondrocytes**

Yu-Hsiu Chen^1,2,5^, Xin Zhang^1,3^, David Attarian MD^3^, Virginia Byers Kraus^1,3,4,5^

^1^ Duke Molecular Physiology Institute, Duke University, Durham, NC, USA

^2^ Division of Rheumatology/Immunology/Allergy, Department of Internal Medicine

Tri-Service General Hospital, National Defense Medical Center

^3^ Department of Orthopaedic Surgery, Duke University, Durham, NC, USA

^4^ Department of Medicine, Duke University School of Medicine, Durham, NC, USA

^5^ Department of Pathology, Duke University Medical Center, Durham, NC, USA

Corresponding Author: Virginia Byers Kraus

Address: 300 N Duke St, Durham, NC 27701

Email: vbk@duke.edu

Key Words CBX4, cellular senescence, DPP4, senomorphic, osteoarthritis

Abstract: 309 words

Main text: 4027 (including table)

**Table S1**. qPCR custom microarray panel (Qiagen, 330171).

| **Gene Symbol** | **Assay Catalog #** | **Gene Symbol** | **Assay Catalog #** | **Gene Symbol** | **Assay Catalog #** |
| --- | --- | --- | --- | --- | --- |
| GDC | PPH65835A | PPC | PPX63339A | RTC | PPX63340A |
| B2M | PPH01094E | YWHAZ | PPH01017A | 18SrRNA | PPH05666E |
| PVRL4 | PPH09678B | PRODH | PPH00877A | LY6D | PPH19736C |
| DAO | PPH11264A | EPN3 | PPH13321A | SLC52A1 | PPH11141A |
| BAX | PPH00078B | BCL2 | PPH00079B | MDM4 | PPH00875E |
| MDM2 | PPH00193E | FAS | PPH00141B | TP53 | PPH00213F |
| TP63 | PPH01032F | CDK1 | PPH00116C | CDK4 | PPH00118F |
| CDKN1A | PPH00211E | CDKN2A | PPH00207C | ATM | PPH00325C |
| STAT1 | PPH00811C | STAT3 | PPH00708F | NFKB1 | PPH00204F |
| TNF | PPH00341F | IL6 | PPH00560C | CXCL8 | PPH00568A |
| HDAC1 | PPH01735F | RB1 | PPH00228F | E2F3 | PPH00917F |
| E2F1 | PPH00136G | E2F7 | PPH19766A | SUMO1 | PPH00973F |
| CSNK2A2 | PPH02197F | DNMT1 | PPH01055F | SOX2 | PPH02471A |
| PARP1 | PPH00686B | MYC | PPH00100B | RING1 | PPH14334B |
| BMI1 | PPH57778A | CBX4 | PPH19160A | DPP4 | PPH00035B |
| SIRT1 | PPH02188A | PCNA | PPH00216B | ATR | PPH01318B |

Supplementary Figure


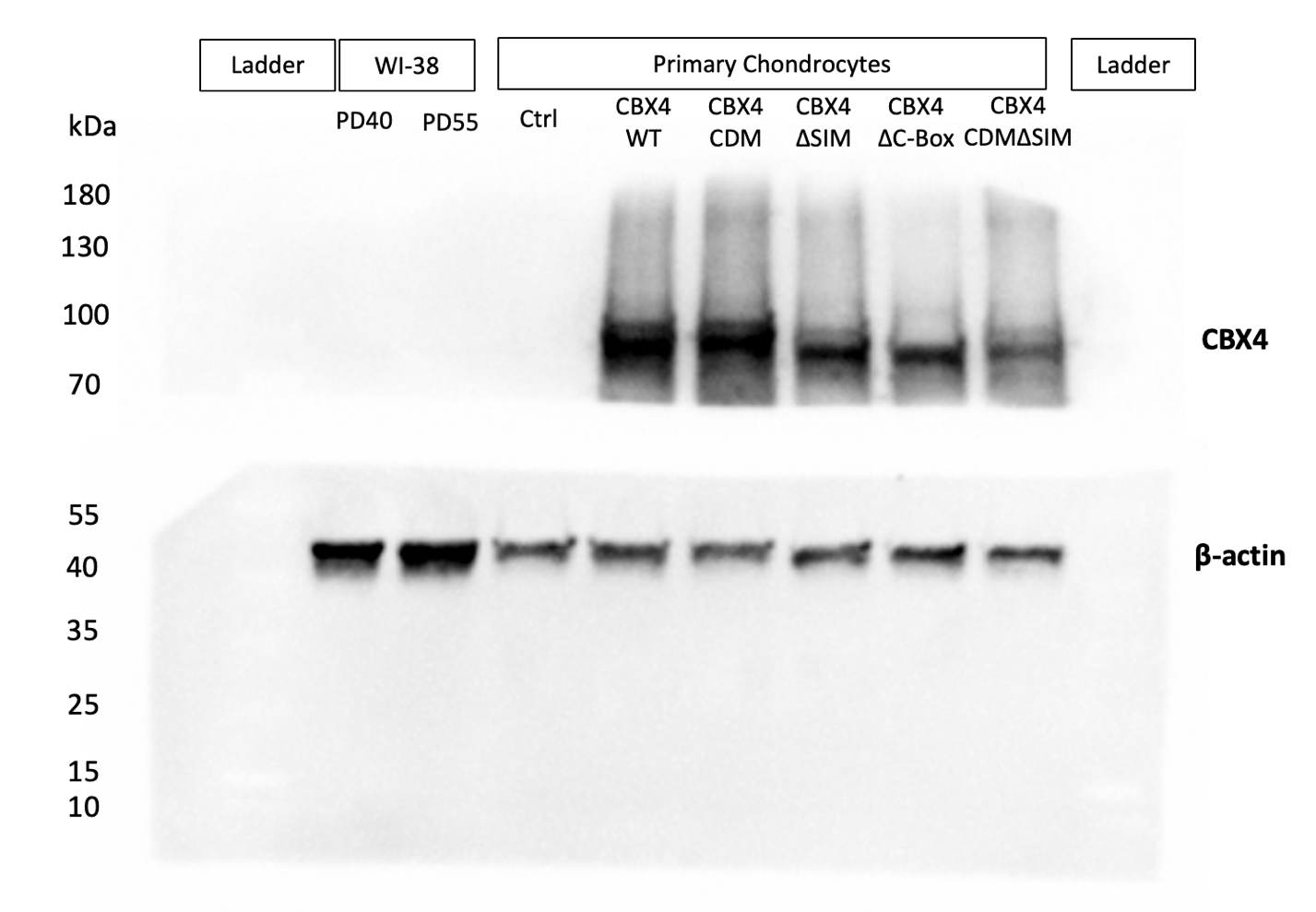


Supplementary Figure 1. "The Western blot underwent a selective exposure with a cutoff at 70 kDa, reflecting substantial variations in protein expression levels between CBX4 and β-actin. In Figure 1A, the blot was further tailored to highlight regions specific to the Control (Ctrl) and CBX4 Wild Type (WT) samples; Figure 2B, focused on CBX4 WT and its mutants within the molecular weight range of 70-100 kDa for CBX4 and 40-55 kDa for β-actin."
